# Elevated Plasma Oligomeric Amyloid β-42 Is Associated with Cognitive Impairments in Cerebral Small Vessel Disease

**DOI:** 10.3390/bios13010110

**Published:** 2023-01-07

**Authors:** Wensheng Qu, Liding Zhang, Xiaohan Liang, Zhiyuan Yu, Hao Huang, Jing Zhao, Yinping Guo, Xirui Zhou, Shabei Xu, Haiming Luo, Xiang Luo

**Affiliations:** 1 Neurological Department, Tongji Hospital, Tongji Medical College, Huazhong University of Science and Technology, Wuhan 430030, China; 2 Hubei Key Laboratory of Neural Injury and Functional Reconstruction, Huazhong University of Science and Technology, Wuhan 430030, China; 3 Britton Chance Center for Biomedical Photonics, Wuhan National Laboratory for Optoelectronics, Huazhong University of Science and Technology, Wuhan 430070, China; 4 MoE Key Laboratory for Biomedical Photonics, School of Engineering Sciences, Huazhong University of Science and Technology, Wuhan 430070, China

**Keywords:** cerebral small vessel disease, sandwich ELISA, amyloid β-42, oligomeric amyloid β-42, white matter hyperintensities

## Abstract

Due to the heterogeneity of amyloid β-42 (Aβ_42_) species, the potential correlation between plasma oligomeric Aβ_42_ (oAβ_42_) and cognitive impairments in cerebral small vessel disease (CSVD) remains unclear. Herein, a sandwich ELISA for the specific detection of Aβ_42_ oligomers (oAβ_42_) and total Aβ_42_ (tAβ_42_) was developed based on sequence- and conformation-specific antibody pairs for the evaluation of plasma samples from a Chinese CSVD community cohort. After age and gender matching, 3-Tesla magnetic resonance imaging and multidimensional cognitive assessment were conducted in 134 CSVD patients and equal controls. The results showed that plasma tAβ_42_ and oAβ_42_ levels were significantly elevated in CSVD patients. By regression analysis, these elevations were correlated with the presence of CSVD and its imaging markers (i.e., white matter hyperintensities). Plasma Aβ_42_ tests further strengthened the predictive power of vascular risk factors for the presence of CSVD. Relative to tAβ_42_, oAβ_42_ showed a closer correlation with memory domains evaluated by neuropsychological tests. In conclusion, this sensitive ELISA protocol facilitated the detection of plasma Aβ_42_; Aβ_42_, especially its oligomeric form, can serve as a biosensor for the presence of CSVD and associated cognitive impairments represented by memory domains.

## 1. Introduction

Cerebral small vessel disease (CSVD) is common in the elderly, with an estimated prevalence of ~5% in 50-year-olds that increases sharply to almost 100% in 90-year-old individuals [1]. CSVD is a disease that primarily affects small arteries, arterioles, and venules in the brain, and causes up to 25% of stroke cases, 45% of dementia cases, and other CNS prevalent disorders [2,3]. Recent advances in neuroimaging, including Magnetic Resonance Imaging (MRI), have provided researchers with the opportunity to detect CSVD by allowing the visualization of neuronal features including white matter hyperintensities (WMH) of presumed vascular origin, lacunes of presumed vascular origin, perivascular Virchow–Robin spaces (PVS), and cerebral microbleeds (CMB) [4].

In addition to imaging characteristics, plasma biomarkers may also help to determine the presence and disease burden of CSVD. Using commercial enzyme-linked immunosorbent assay (ELISA) kits, some reports suggested that higher plasma levels of amyloid β (Aβ) are associated with CSVD. In the RUN DMC study, elevations of plasma Aβ_38_, Aβ_40_ and Aβ_42_ appeared, which were associated with imaging markers cross-sectionally and longitudinally [5]; in the Rotterdam study, higher levels of plasma Aβ_38_, Aβ_40_ and Aβ_40_/Aβ_42_ ratio in CSVD patients were related to worse performance on cognitive tests specifically in the memory domain [6]. However, in the three-City Dijon Study, low plasma Aβ_40_ and Aβ_42_ levels were associated with accelerated progression of WMH over the follow-up of dementia-free older persons [7]. Since the discrepancies exist, it is of great significance to research the correlations between Aβ isoforms and CSVD in more cohorts, thereby determining the potential plasma biomarkers for cognitive impairments in CSVD.

Among the isoforms of Aβ, Aβ_42_ is considered an early contributor to Alzheimer’s disease (AD) pathology [8]. Aβ_42_ peptides are detected as monomers (mAβ_42_) and soluble oligomers (oAβ_42_). oAβ_42_ is the main toxic subspecies, which triggers a cascade of downstream injuries to cause neuronal dysfunction and death [9,10]. We hypothesized that oAβ_42_ might be a sensitive biosensor for CSVD. However, no relevant reports have been published and its reliable detection is still a challenge [11,12,13,14]. Transformed after monomer aggregation, oAβ_42_ has variable subspecies ranging from low (LMW) to high molecular weight (HMW) soluble protofibril (pAβ_42_); its presence is usually transient and heterogeneous and dynamic equilibrium varies between mAβ_42_ and oAβ_42_. Traditional detection methods mainly target the reduced Aβ_42_ rather than the elevated oAβ_42_, which caused inconsistent results of Aβ_42_ levels in plasma [15,16,17,18,19]. Recently, several diagnostic tools including ELISA [20], aptamer sensor [21], fluorescence [22], surface-enhanced Raman scattering (SERS) [23], linear sweep voltammetry [24], and differential pulse voltammetry [25] were developed, but most of them failed to meet clinical accuracy (Table 1). ELISAs have good accuracy and low limits; however, the Aβ-based ELISA prepared currently are partially selected from Aβ conformations such as pAβ and oAβ. We previously developed a sandwich ELISA for the specific detection of different soluble Aβ_42_ species based on a pair of anti-Aβ antibodies with different epitopes, which successfully detected mAβ_42_, oAβ_42_, and/or total Aβ_42_ (tAβ_42_) [26,27].

In the present study, we utilized this well-performing ELISA, combining with MR imaging and neuropsychological evaluations, to evaluate the associations between plasma Aβ_42_, imaging characteristics of CSVD, and cognitive subdomains in a Chinese CSVD community cohort. We hope that the analysis of Aβ_42_ subspecies, especially the oAβ_42_, may contribute to the early diagnosis of CSVD and further the understanding of CSVD pathogenesis.

## 2. Materials and Methods

### 2.1. Study Populations

This cross-sectional prospective observable case-control study was based on ongoing research in the Tongji-CSVD community cohort, which is investigating the prevention and diagnosis of sporadic CSVD. Candidates aged 55–85 were recruited from communities in Wuhan city. Participants must have presented without non-vascular white matter lesions, neurodegenerative diseases, acute cerebral vascular diseases, large cerebral vessel stenosis, serious psychological disorders, other serious non-vascular CNS diseases such as AD, or systemic diseases. Detailed information is shown in the Chinese Clinical Trial Registry (No. ChiCTR1900027225). This study was approved by the Ethics Committee of Tongji Medical College (No. 2019-S105). All candidates provided informed consent in accordance with the Declaration of Helsinki.

### 2.2. Brain MR Imaging

All participants were scanned using a single 3.0 Tesla scanner (Union imaging, China). The following images were obtained from each patient: T1-weighted, T2-weighted, T2 fluid-attenuated inversion recovery (FLAIR), 3D-TOF MR angiography, diffusion-weighted imaging, and susceptibility-weighted imaging (SWI) sequences. A double-blind evaluation of all images was performed by two trained neurologists to exclude other CNS diseases and to evaluate all the CSVD imaging markers according to the STRIVE definitions [28].

Briefly, WMH was defined as a signal abnormality of variable size in the white matter that exhibits hyperintensity on T2-weighted images without cavitation. The severity of WMH was assessed at six points according to Fazekas’s score [29]. Lacunes of presumed vascular origin were defined and counted as ovoid or round, fluid-filled cerebrospinal fluid (CSF)-cavities in the subcortical areas, with a diameter between 3 and 15 mm, consistent with a previous acute small subcortical infarct or hemorrhage in the territory of one perforating arteriole. A CMB was evaluated using SWI, defined as a focal area (generally 2–5 mm in diameter, but up to 10 mm) of low signal intensity on gradient echo. Visible PVS was identified as linear and round or ovoid spaces following the course of a vessel with a perpendicular diameter < 3 mm and without a hyperintense rim on FLAIR imaging.

### 2.3. Groups

**Figure 1:**
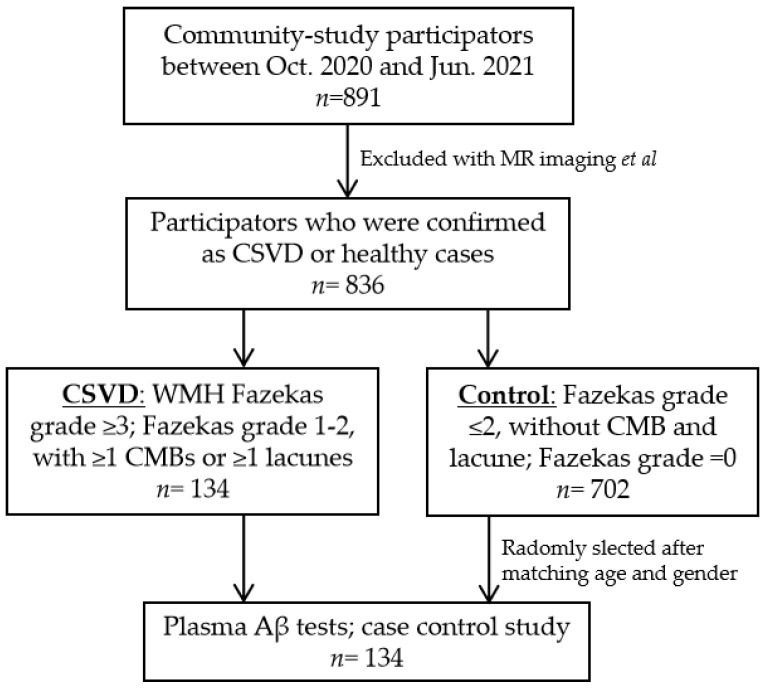
Flow chart for enrollment of participants.

While there is no uniform standard, we identified CSVD using modified standards according to previous reports [30,31] after the exclusion of other CNS diseases. CSVD was defined as the presence of any following items: WMH Fazekas grade ≥3; Fazekas grade 1–2, with ≥1 CMBs or ≥1 lacunes. Since CSVD is of low prevalence and aging-dependent [1], case matching is preferred to reduce variations. Among the participants in this cohort, healthy controls (with WMH Fazekas grade ≤2 only) were randomly selected (1:1) using software (SPSS Inc., Chicago, IL, USA) after matching by age (+/−2 years) and gender (Figure 1). Cerebral amyloid angiopathy (CAA), heritable CSVD, and AD were ruled out according to expert opinions and related imaging characteristics. Extended laboratory investigations such as a CSF test, positron emission tomography (PET), and gene analysis were performed if necessary.

### 2.4. Demographics and Vascular Risk Factors

Demographic variables included age, gender, and years of education. Some commonly established stroke risk factors were determined using the standardized case report form [32] and included obesity, hypertension, diabetes, hyperlipidemia, previous stroke incidences, episodic alcohol consumption (drinking), and smoking. These factors were further classified as positive or negative (i.e., smoking behavior was classified as never vs. former/current smoking; episodic alcohol consumption was defined as >5 alcoholic drinks per day at least once a month; body mass index ≥24 kg/m^2^ indicated obesity; and previous attacks of transient ischemic stroke, ischemia or hemorrhage were considered strokes).

### 2.5. Evaluation of Neuropsychological Function

Cognitive function was assessed using a standardized neuropsychological battery test that is sensitive and suitable for CSVD research [32]. Global cognitive function was evaluated through the Mental State Examination (MMSE) and Montreal Cognitive Assessment (MoCA). The MMSE is a set of 11 questions, with a maximum score of 30. The MoCA probes more cognitive domains, including executive functioning, immediate and delayed memory, visuospatial abilities, attention, working memory, language, and orientation to time and place. Its score ranges from 0 to 30 points.

Cognitive subdomains were tested using the Stroop color word, Rey Auditory Verbal Learning (AVLT), Trail Making (TMT), digit span, Rey Osterrieth complex figure (ROCF), Boston naming, and Clock drawing tests (CDT). The Stroop color word test is based on the observation that subjects can read words much faster than they can identify and name colors. A 45 min test was performed to reflect the cognitive flexibility, resistance to interference from outside stimuli, creativity, and psychopathology. For AVLT, five presentations of a 15-word list were given, followed by attempted recall, delayed recall and recognition. The TMT is comprised of two tasks. Part A consists of 25 circles numbered, while Part B consists of 25 circles numbered 1 to 13 and lettered A to L, randomly distributed. The subject was required to connect the circles as quickly as possible in numerical sequence, in order to measure visual attention and task switching ability. During the Digit Span test, participants were presented with a random series of digits, and were asked to repeat them in either the order presented (forward span) or in reverse (backwards span). The simpler forward span task requires verbal working memory and attention, while the backwards span task additionally tests cognitive control and executive function. The ROCF was developed by André Rey and Paul-Alexandre Osterrieth. Subjects were asked to copy this figure in order to investigate visuospatial constructional functions, visuographic memory and some aspects of planning and executive function. During the BNT, subjects were shown line drawings of 30 common objects one at a time and were asked to name them orally. During the CDT, subjects was asked to draw a clock, which was scored with a maximum of 4. This requires visual-spatial, numerical sequencing, and planning abilities.

### 2.6. The Sandwich ELISA for Plasma Aβ Measurements

The sandwich ELISA prepared for detecting different Aβ_42_ species was based on a pair of sequence- and conformation-specific antibodies as our previously screened [26,27]. For the specific detection of oAβ_42_, single epitope antibody 1F12 and biotinylated 1F12 were used as both capture and detection antibodies. For tAβ_42_, the single epitope antibody 1F12 was used as the capture antibody, and biotinylated 2C6 with four different epitopes was used as the detection antibody. The detailed detection processes were as follows: 96-well plates were coated with 1 μg/well of 1F12 and blocked with 5% skimmed milk for 2 h at 37 °C. Then, plasma samples were added into each well for 2-h incubation at 37 °C, followed by incubation with biotinylated 1F12 (1:1000) or biotinylated 2C6 (1:1000), and streptavidin-coupled poly-HRP (1:8000) for 1 h at 37 °C ordinally. The immunoreaction was visualized with high sensitivity soluble 3,3′,5,5′-Tetramethylbenzidine (TMB, Abcam, Cambridge, UK) substrate solution and analyzed at an absorbance of 450 nm, after being terminated by 2 M H_2_SO_4_. The mAβ_42_ level was calculated by subtracting the oAβ_42_ level from the tAβ_42_ level.

The specificity of the sandwich ELISA was evaluated using several similar non-target blood biomarkers including mAβ_42_, oAβ_42_, mAβ_40_, and oAβ_40_. Two standard oAβ_42_ and tAβ_42_ protein solutions ranging from 500 pM to 3.9 pM and 77 pM to 0.6 pM were used for the analysis of sensitivity. The sandwich ELISA was performed as the above testing protocol.

Whole blood samples with EDTA anticoagulant were collected along with MR imaging and were stored at −80 °C in Wuhan Biobank, Hubei, China. Blood samples were thawed immediately before Aβ quantification. The plasma levels of tAβ_42_ and oAβ_42_ were measured using a prepared sandwich ELISA as described above. Briefly, 100 μL of each plasma sample was added into antibody 1F12 coated 96-well plates and incubated for 2 h at 37 °C. After washing three times with PBS-T, the plates were incubated with detector antibody (biotinylated 2C6 for tAβ_42_ detection or biotinylated 1F12 for oAβ_42_ detection) for 1 h, followed by 1 h incubation with streptavidin-coupled poly-HRP and 20 min incubation with high sensitivity soluble TMB, substrate solution for a chromogenic reaction. Finally, the reaction was stopped and the absorbance of each well was read at 450 nm with a microplate reader. All the incubation steps were performed in a shaker with 60 rpm at 37 °C. The operators were trained with a familiarization panel immunosorbent assay to get acquainted with the assay protocol and sample manipulation.

### 2.7. Statistical Analysis

The SPSS 20.0 software package was used for data management and analyses. A *p*-value < 0.05 was considered statistically significant. The normal distribution was assessed with the Kolmogorov–Smirnov test. For intergroup comparisons, the chi-square test was applied for categorical variables, and results were shown as percentages; the Mann–Whitney *U* test was performed for continuous variables without normal distribution, and results were presented as medians [interquartile ranges 25–75%]; the unpaired Student’s *t*-test was used for continuous variables with normal distribution, and results were expressed as Mean standard ± deviation (SD); the one-way analysis of variance (ANOVA) was used for multigroup comparisons, followed by Tukey’s post hoc test for between groups.

For data from all participants, Spearman’s rank correlation was performed to show the correlation between tAβ_42_ and oAβ_42_. To measure the associations of risk factors with the presence of CSVD and its imaging markers, WMH was divided into limited (Fazekas score ≤ 2) and serious (Fazekas score 3–6) conditions. Other imaging markers were considered present when: PVS ≥ 11, CMB ≥ 1, and lacune ≥ 1. Aβ_42_ and neuropsychological scores alike were divided by quintiles. Binary or ordinal logistic regression was performed after adjustment for associated variables. Odds ratios (ORs) with their 95% confidence intervals (CIs) were obtained.

To evaluate the possible predictive value for the presence of CSVD, receiver operating characteristic (ROC) curves were plotted and the area under the ROC curve (AUC) was calculated. *P*-trends for continuous linear trends per stratum were analyzed using the Mantel–Haenszel chi-square test. Compound domain scores were calculated from the Z-scores of each neuropsychological test. Linear regression was performed after averaging the Z-scores within each domain. Tests to assess the memory domain included AVLT (immediate recall, delayed recall, and recognition) and Digit Span Forward.

## 3. Results

### 3.1. Assay Principle of the Proposed Sandwich ELISA

**Figure 2:**
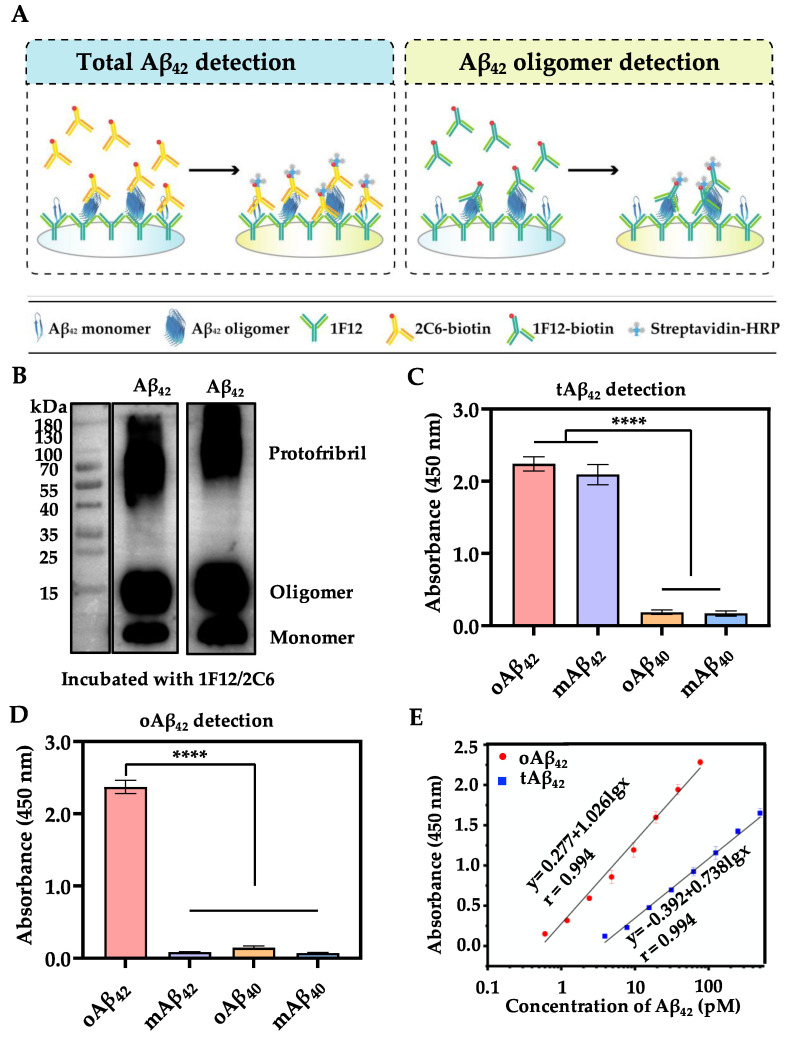
The performance of sandwich ELISA. (**A**) Principle of the proposed sandwich ELISA for tAβ_42_ and oAβ_42_ detection. (**B**) Western blotting analysis of the prepared Aβ_42_ species using antibody 1F12 or 2C6. The specificity assay in tAβ_42_- (**C**) and oAβ_42_- (**D**) sandwich ELISA. (**E**) The plotted linear curve of different oAβ_42_ and tAβ_42_ conformations. Data are presented as means ± SD, *n* = 3. One-way analysis of variance (ANOVA) was used for multigroup comparisons. Statistical significance is represented in the figures by **** *p* < 0.0001.

For the accurate and specific detection of different forms of Aβ_42_, combinations of antibody pairs were screened. tAβ_42_ detection is based on the antibody pair 1F12/2C6 with different epitopes, of which 1F12 has single epitope, while 2C6 has four epitopes in mAβ_42_; this ensured that both mAβ_42_ and oAβ_42_ could be detected (Figure 2A, left). On the contrary, a similar and single epitope antibody pair 1F12/1F12 was selected for oAβ_42_ detection to avoid mis-detecting mAβ_42_, due to the epitope competition between the 1F12/1F12 and mAβ_42_. oAβ_42_ was aggregated by several mAβ_42_ exposing multiple identical epitopes, which could be recognized by its corresponding and specific epitope antibody pair 1F12/1F12 (Figure 2A, right). Such a strategy allows the dimers and above Aβ_42_ aggregates to be detected, but not the mAβ_42_. 

To our knowledge, this is a new report to distinguish exactly between all forms of soluble tAβ_42_ and oAβ_42_ (Table 1).

**Table 1:** Comparison of sandwich ELISA with other detection methods.

Method	Target	Specificity	Sensitivity	Ref.
ELISA	tAβ_42_, oAβ_42_, mAβ_42_	High	3.9, 0.6 pM	Current method
ELISA	pAβ_42_	High	< 0.68 pM	[20]
MSD	oAβ_42_	No	1.5 pM	[33]
TES	oAβ_42_,	NA	4 pM	[34]
	fAβ_42_			
Simoa	oAβ_42_	High	0.22 nM	[35]
Aptamer	oAβ_42_	Moderate	12.5 nM	[21]
SERS	oAβ_42_	No	10 nM	[23]
SWV	oAβ_42_	No	48 pM	[36]
LSV	oAβ_42_	High	8 pM	[24]
DPV	oAβ_42_	High	0.1 nM	[25]
FL	oAβ_42_	High	0.2 nM	[22]

Abbreviations: DPV, differential pulse voltammetry; ELISA, enzyme-linked immunosorbent assay; FL, fluorescence; LSV, linear sweep voltammetry; MSD, Meso Scale Discovery; Simoa, Single Molecular Array; SWV, square wave voltammetry; SERS, surface-enhanced Raman spectroscopy; TES, two-channel electrochemical system; t/o/p/f mAβ_42_, total/oligomeric/protofibril/fibril amyloid β-42.

### 3.2. Characterization of Sandwich ELISA

The performance of sandwich ELISA relies deeply on antibody pairs. We first evaluated whether the antibody 1F12 and 2C6 can recognize different Aβ_42_ aggregates. It was demonstrated that both 1F12 and 2C6 reacted well with mAβ_42_, LMW-oAβ_42_, and HMW-pAβ_42_, indicating that these two antibodies can recognize all soluble Aβ_42_ species and are powerful tools for the measurement of Aβ_42_ (Figure 2B). Then, the performance of sandwich ELISA was evaluated and the results showed that, in the antibody pair 1F12/2C6, both mAβ_42_ and oAβ_42_ can be detected and showed no cross-reaction with oAβ_40_ and mAβ_40_ (Figure 2C). Similarly, oAβ_42_ can be specifically recognized by the antibody pair 1F12/1F12, rather than the mAβ_42_, oAβ_40_, and mAβ_40_ (Figure 2D). These data reflected that the established oAβ_42_/tAβ_42_-sandwich ELISA had a great selective specificity. We observed two strong linear correlations between the absorbance change and the biomarkers concentration in the range of 77 to 0.6 pM for oAβ_42_ and 500 to 3.9 pM for tAβ_42_ (Figure 2E). Based on the results of linear regression analysis, the limit of quantification of the sandwich ELISA was estimated to be 0.6 pM for oAβ_42_ and 3.9 pM for tAβ_42_, respectively.

### 3.3. Clinical Characteristics

In this study, 134 participants aged 56–83 years were enrolled in each group. The clinical characteristics of study participants are presented in Table 2. Compared with controls, CSVD patients had a significantly higher prevalence of hypertension and previous stroke incidences but were generally less educated. Binary logistic regression evaluation of variables including age, gender, obesity, diabetes, hyperlipidemia, drinking, and smoking showed that hypertension and previous stroke incidences are independent risk factors for the presence of CSVD (Table 3).

### 3.4. Plasma Aβ_42_ and CSVD

**Figure 3:**
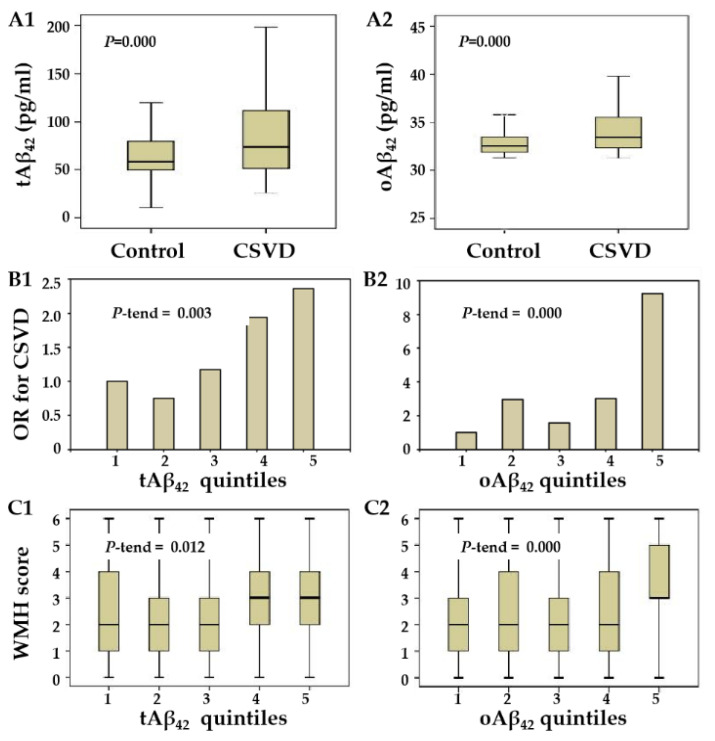
Plasma Aβ_42_ levels and the correlations with CSVD/WMH presence. Relative to that in the control group, either the tAβ_42_ (**A1**) or oAβ_42_ (**A2**) level was elevated in the CSVD group. Correlations between the presence of CSVD and t/oAβ_42_ quintiles were analyzed by logistic regression analyses after adjustment for age and sex. *P*-trends for continuous linear trend per stratum were calculated by Mantel–Haenszel chi-square tests and are displayed in the upper left corners. ORs of quintiles for CSVD are presented in (**B1**,**B2**); Grouped by t/oAβ_42_ quintiles, WMH scores are displayed in Box-and-whisker plots (**C1**,**C2**).

**Figure 4:**
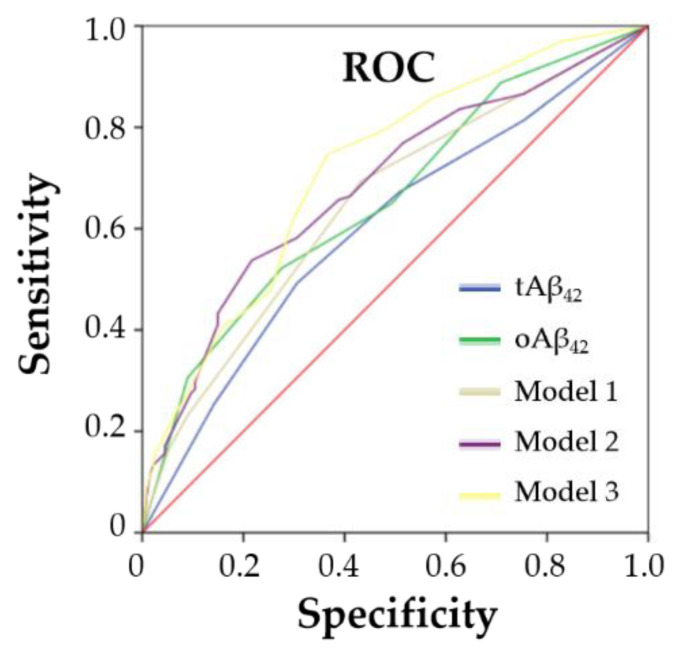
AUC for predicting the presence of CSVD. Model 1: CSVD = −0.594 + 0.865 × hypertension + 0.990 × previous stroke; Model 2: CSVD = −1.343 + 0.254 × tAβ42 + 0.864 × hypertension+ 0.974 × previous stroke; Model 3: CSVD = −1.785 + 0.405 × oAβ42 + 0.864 × hypertension+ 0.947 × previous stroke.

A positive correlation was evident between tAβ_42_ and oAβ_42_ (*r* = 0.317, *p* = 0.000). Either tAβ_42_ or oAβ_42_ was much higher in the CSVD group compared with that in the control group (Figure 3A, Table 2). A linear increase in CSVD prevalence appeared depending on elevated plasma Aβ_42_ quintiles (*p*-trend < 0.05) (Figure 3B). In particular, relative to the lowest quintile, participants with the highest quintile of plasma oAβ_42_ had a much higher risk of CSVD (9.229 [3.811–22.349]; *p* = 0.000). Furthermore, the elevation of either tAβ_42_ or oAβ_42_ quintiles correlated with CSVD presence when adjusted by age, gender, previous stroke, and hypertension (Table 3), suggesting that tAβ_42_ or oAβ_42_ may be independent plasma sensors for the presence of CSVD. Calculations show that the AUC for tAβ_42_ was 0.625 [0.559–0.692] and 0.616 [0.549–0.683] for oAβ_42_. Compared to the model using hypertension and previous stroke, Aβ_42_ contributed to a slightly improved discriminatory accuracy of the whole model (the AUC was elevated by tAβ_42_ from 0.651 to 0.693, while the oAβ_42_ elevated it to 0.699) (Figure 4, Table S1 in Supplementary Materials).

After adjusting for gender, age, and all observed vascular risk factors, the elevation of Aβ_42_ quintiles correlated with the presence of severe WMH, whereas the elevated oAβ_42_ quintiles also correlated with the CMB (Table 4). As suggested by *P*-trend, a linear increase in the prevalence of severe WMH appeared to be dependent on elevated plasma Aβ_42_ quintiles (Figure 3C). In particular, WMH scores were much higher in the highest quintile of plasma oAβ_42_, compared with the lowest quintile.

### 3.5. Plasma Aβ_42_ and Cognition

Neuropsychological impairments were more prevalent in CSVD cases compared to controls (Table S2). It was no surprise that CSVD participants reported impaired global cognitive function, impaired visuospatial function, reduced information processing speed, impaired executive function, impaired speech, lesioned memory domain, and elevated Hamilton depression/anxiety scores (*p* < 0.05).

After adjusting for age, gender, education, and Hamilton depression/anxiety scores, elevated tAβ_42_ quintiles correlated with impaired executive domain according to the digit span test and short-delayed recall domain of AVLT. Elevated oAβ_42_ quintiles correlated with delayed recall and recognition domains of AVLT (Table 5). Predictors of composite scores in the memory domain were investigated using variables including oAβ_42_ quintiles, gender, age, education, and Hamilton depression/anxiety scores with a linear regression test. Indeed, oAβ_42_ quintiles (β = −0.113 [−0.173–0.053], *p* = 0.000), education (*p* = 0.000), and age (*p* = 0.015) were all associated with composite scores of memory domain (R^2^ = 0.258, *p* = 0.000).

## 4. Discussion

Although several detection methods for the measurement of Aβ_42_ have been reported previously, most are of partial selectivity for conformations such as oAβ_42_, pAβ_42_ or fAβ_42_, listed in Table 1. Accumulating evidence strongly supports that soluble oligomeric Aβ_42_, ranging from LMW to HMW, is neurotoxic [37,38]. Therefore, the accurate detection of oligomers with different molecular weights but not the mAβ_42_ is of great significance for the diagnosis and deep understanding of the pathology. To date, accurate differentiation of all forms of oAβ_42_ from tAβ_42_ remains a challenge, because it suffers from the mis-detection of mAβ_42_ and lacks a pair of conformation-specific antibodies. Previously developed Aβ-based diagnosis tools were mainly based on a commercial antibody 6E10 reacting with almost all forms of Aβ at the N terminus but not oAβ_42_ and pAβ_42_ [39,40,41,42], or used conformation-specific antibodies such as mAb 7A1 (reacting with oAβ but cross-reacting with Aβ_40_) [33], mAb 158 (specific reacting with pAβ_42_) [20], and mOC64 (specific reacting with fAβ_42_) [42]. The above-described antibodies cannot detect either oAβ_42_ subspecies or tAβ_42_. Thus, we developed a sandwich ELISA for the precise and accurate analysis of different forms of Aβ_42_ including mAβ_42_, oAβ_42_ subspecies ranging from LMW to HMW, and tAβ_42_, which addressed the challenge of the current plasma Aβ_42_ detection and made it possible to research the correlations between Aβ_42_ and CSVD.

Based on the results of this sandwich ELISA, our findings showed that the elevated plasma tAβ_42_ and oAβ_42_ appeared in CSVD cases and was associated with the presence of CSVD imaging makers such as WMH and CMB. Notably, both tAβ_42_ and oAβ_42_ are candidates to be plasma biosensors for CSVD. The elevation of Aβ_42_, especially oAβ_42_, was further correlated with cognitive impairments, as represented by the memory domain, which usually appeared in CSVD.

Elevated Aβ deposition is commonly seen in AD and CAA. The pathology of AD and CAA intersects at the increased Aβ generation and impaired Aβ clearance [43]. However, whether Aβ is a pathogenic factor for sporadic CSVD remains unclear. Autopsy studies have shown that brain Aβ levels are frequently elevated among CSVD patients with clinical AD [44]. Brain Aβ is also associated with higher WMH volume in individuals with vascular dementia, as measured by PET [45]. Finally, a stronger association exists between arteriolosclerosis and cortical microinfarcts with greater Aβ brain deposition as identified using immunohistochemistry [46].

In contrast, although brain Aβ was associated with CSVD burden cross-sectionally, CSVD does not appear to directly influence Aβ accumulation longitudinally, as shown after 1-year follow-up in the elderly [47]. A thorough review of 34 reports from January 2000 to September 2015 demonstrated no significant relationships between Aβ and WMH burden [48]. Aβ can also be detected in CSF, and young dementia patients; the prevalence of CSVD is highly concomitant with the prevalence of low CSF Aβ_42_ [49]. However, the Gothenburg Mild Cognitive Impairment study showed that CSF Aβ_42_ does not necessarily change in patients with subcortical CSVD despite reduced CSF levels of soluble APP-β [50].

With the development of high-sensitivity assays, Aβ can now be detected in plasma in addition to brain tissue and CSF. Several recent studies have shown that plasma Aβ may be a promising early biomarker for the ongoing amyloid pathology in AD [51,52,53]. However, mixed results have been reported regarding the correlation between plasma Aβ and AD. Systemic studies showed that plasma Aβ alone weakly predicts the development of AD and dementia [54]. In the RUN DMC study, elevated plasma Aβ was associated with the presence and progression of CSVD markers such as CMB, lacunes, and severe WMH, although participants with incident CMB had elevated plasma Aβ_42_ levels [5]. In the Rotterdam Study, higher plasma levels of Aβ levels were also associated with CSVD markers and poorer memory. In particular, higher levels of Aβ_40_/Aβ_42_ were significantly associated with increased lacunar and microbleed counts, as well as increased WMH volume and poorer cognition [6]. However, low plasma Aβ_42_ was associated with a higher risk of extensive WMH progression over the follow-up of dementia-free older persons in the three-City Dijon Study [7]. Here, the Chinese sporadic CSVD cohort demonstrated elevated plasma Aβ_42_ levels in CSVD cases. In particular, these levels were significantly correlated with the presence of severe WMH. Hence, as one independent predictor for the presence of CSVD, testing plasma Aβ_42_ levels might enhance the predictive power of historic risk factors, albeit with compromised performance.

It is well known that either Aβ or CSVD is correlated with cognitive impairment in the elderly. A moderately significant correlation between brain fibrillar amyloid load and episodic associative memory tasks has been reviewed elsewhere [55]. The increasing early and intense Aβ deposition leads to the discoordination of neural networks that predict behavioral performance across a series of cognitive tasks, including attention, working memory, and episodic memory [56,57]. Meta-analyses have indicated that plasma Aβ_40_/Aβ_42_ ratio predict the development of AD-related dementia, despite significant heterogeneity [54]. Some data also suggest that a higher Aβ_40_ level is associated with poorer memory and information processing speed, as well as cognitive decline in cognitively normal adults [58]. For CSVD, the Rotterdam Study showed that higher levels of both Aβ_38_ and Aβ_40_, as well as the Aβ_40_/Aβ_42_ ratio, are associated with worse memory domains of AVLT in asymptomatic older adults [6]. Our study suggests a weak but significant correlation between plasma Aβ_42_ and cognitive domains. Digit span forward and backward tests, as well as the AVLT, showed that worse memory domains were correlated with increased plasma Aβ_42_, particularly oAβ_42_.

Mounting evidence indicates that soluble oAβ_42_, the most toxic species, can effectively induce neuronal death [59]. Previously, the detection of plasma Aβ oligomers was challenging due to their low abundance and heterogeneity. With a modified assay, elevated plasma Aβ oligomers have been found to correlate with decreased MMSE in AD patients, which can be used to evaluate brain amyloid deposition [60]. Aβ oligomers are also considered useful biomarkers for AD diagnosis [60,61]. While Aβ oligomers may be useful for diagnosing AD [59], their potential diagnostic value in CSVD patients remains unknown. The present results indicate that elevated plasma oAβ_42_ correlates with CSVD and the impairments of memory domains reflected by digit span and AVLT. As reported, the plasma concentrations of Aβ_42_ are unstable partly because of the transformation, and they vary in individuals depending on gene background [62]. Relative to the obvious variation of tAβ_42_ individually, oAβ_42_ was stably detected here. This supports that oAβ_42_ is more likely to be a plasma indicator for CSVD. As a subtype of CSVD, CAA has been known to closely correlate with Aβ deposition [63]. CAA would be of interest for researching the functions of oAβ_42_ in vascular dementia.

There are several limitations to the current study. First, because CSVD presents age-dependently, we selected matching controls from community populations, which could lead to potential biases. Second, other historic factors including involvement in sports, prior medication, and diet were not considered. Genetic tests to confirm the sporadic background were also not performed totally, and it remains uncertain whether AD was concomitant in the cohort only from MR imaging and clinical follow-up. Third, although positive results are described, further investigations with increased sample size are necessary to gain greater reliability. Deeper research using animal models or other multimode imaging would be beneficial. Future steps to assess causality, predictive models, and interactions between biosensors and CSVD would also be preferred.

## 5. Conclusions

In the present study, plasma Aβ_42_ was associated with the presence of CSVD and cognitive impairments represented by memory domains. Testing plasma Aβ_42_, especially oAβ_42_, may be helpful for closely monitoring CSVD-related CNS damage. With continued investigation, oAβ_42_ may have the potential to be a plasma biomarker for the presence of CSVD and related cognitive impairments.

## Figures and Tables

**Table 2:** Clinical characteristics and plasma Aβ_42_ levels between groups.

	Control (*n* = 134)	CSVD (*n* = 134)	*p*
Age (y)	67.0 ± 6.1	67.5 ± 6.1	0.994
Male (%)	70 (52.2)	70 (52.2)	1.000
Obesity (%)	65 (48.5)	71 (53.0)	0.463
Hypertension (%)	53 (39.6)	84 (62.7)	0.000*
Diabetes (%)	28 (20.9)	32 (23.9)	0.558
Hyperlipemia (%)	31 (23.1)	33 (24.6)	0.774
Smoke (%)	31 (23.1)	33 (24.6)	0.774
Drinking (%)	28 (20.9)	31 (23.1)	0.658
Previous stroke (%)	12 (9.0)	35 (26.1)	0.000 *
Education years (y)	12.0 [9.0–13.3]	9.0 [9.0–12.0]	0.023 *
tAβ_42_ (pg/mL)	58.32 [49.22–79.80]	73.47 [51.75–112.03]	0.000 *
oAβ_42_ (pg/mL)	32.58 [31.89–33.49]	33.44 [32.33–35.53]	0.000 *

Intergroup comparisons were performed by chi-square test, Mann-Whitney U test, or unpaired Student’s *t*-test. * *p* < 0.05.

**Table 3:** Independent factors for the presence of CSVD.

	Factors	OR [95%CI]	*p*
History	gender	0.819 [0.434–1.543]	0.536
	hyperlipidemia	0.839 [0.456–1.542]	0.571
	diabetes	0.947 [0.500–1.793]	0.866
	age	1.005 [0.964–1.049]	0.803
	obesity	1.002 [0.601–1.669]	0.995
	smoke	1.017 [0.485–2.133]	0.964
	drinking	1.220 [0.568–2.620]	0.611
	Previous stroke	2.692 [1.307–5.545]	0.007 *
	hypertension	2.374 [1.439–3.917]	0.001 *
Plasma tests ^†^	tAβ_42_ quintiles	1.292 [1.080–1.546]	0.005 *
	oAβ_42_ quintiles	1.500 [1.242–1.811]	0.000 *

Binary logistic regression, CSVD, and control groups were matched by age and gender. ^†^ Adjusted by age, gender, previous stroke, and hypertension; * *p* < 0.05.

**Table 4:** Correlations of plasma Aβ_42_ levels to CSVD imaging markers.

		WMH	Lacune	CMB	PVS
tAβ_42_ quintiles	OR [95%CI] P	1.336 [1.138–1.640] 0.001 *	1.202 [0.960–1.506] 0.109	1.204 [0.954–1.518] 0.118	1.156 [0.917–1.457] 0.221
oAβ_42_ quintiles	OR [95%CI] *p*	1.481 [1.228–1.786] 0.000 *	1.163 [0.928–1.457] 0.191	1.358 [1.068–1.727] 0.012 *	0.900 [0.713–1.134] 0.371

Binary logistic regression. Adjusted by age, gender, obesity, previous stroke, hypertension, hyperlipidemia, diabetes, drinking, and smoking. * *p* < 0.05.

**Table 5:** Correlations of plasma Aβ_42_ levels to cognitive subdomains.

		Digit Span-Forward	Digit Span-Backward	AVLT-n4	AVLT-n5	AVLT-n6
		Short-Term Memory	Working Memory	Short-Delayed Recall	Long-Delayed Recall	Recognition
tAβ_42_ quintiles	OR 95%CI *p*	0.998 [0.996–1.001] 0.146	0.996 [0.992–1.000] 0.036 *	0.890 [0.792–1.000] 0.049 *	1.000 [0.999–1.002] 0.682	1.002 [1.000–1.004] 0.050
oAβ_42_ quintiles	OR 95%CI *p*	0.970 [0.932–1.009] 0.124	0.974 [0.935–1.015] 0.215	0.951 [0.912–0.991] 0.017 *	0.943 [0.905–0.982] 0.005 *	0.950 [0.913–0.989] 0.013 *

Ordinary regression analysis. Adjusted by age, gender, education years, Hamilton depression, and anxiety. * *p* < 0.05.

## Data Availability

Data presented in this study are available on request from the corresponding author. The data are not publicly available due to restrictions of by privacy policies.

## Supplementary Materials

The following supporting information can be downloaded at: https://www.mdpi.com/article/10.3390/bios13010110/s1, Table S1: AUC to predict the presence of CSVD; Table S2: Results of neuropsychological tests between groups.
