# Supplementary material for: Elevated Plasma Oligomeric Amyloid β-42 Is Associated with Cognitive Impairments in Cerebral Small Vessel Disease
Source: Biosensors (Basel). 2023 Jan 7;13(1):110. doi: 10.3390/bios13010110 (PMC9855662; doi:10.3390/bios13010110)
Supplement: Supplementary file 1 [file biosensors-13-00110-s001.zip › biosensors-2067564-supplementary.pdf]

Table S1: AUC to predict the presence of CSVD

Predictor	AUC	95% CI	sensitivity	specificity
$tA\beta_{42}$	0.625	0.559–0.692	49.3%	69.4%
$oA\beta_{42}$	0.616	0.549–0.683	55.2%	72.4%
Model 1	0.651	0.586–0.717	69.4%	56.7%
Model 2	0.693	0.630–0.757	53.7%	78.4%
Model 3	0.699	0.636–0.761	74.6%	63.4%

Model 1: CSVD=−0.594+ 0.865* hypertension+ 0.990* previous stroke

Model 2: CSVD=−1.343+ 0.254* $tA\beta_{42}$+ 0.864* hypertension+ 0.974* previous stroke

Model 3: CSVD=−1.785+ 0.405* $oA\beta_{42}$ + 0.864* hypertension+ 0.947* previous stroke

CSVD, cerebral small vessel disease; t/$oA\beta_{42}$, total/ oligomeric amyloid beta-42; OR, odds ratios; ROC, receiver operating characteristic curves; AUC, the area under the ROC curve; CI, confidence interval.

Table S2: Results of neuropsychological tests between groups

	CSVD	Control	Z	P
MMSE	28.0 [26.0–29.0]	28.5 [27.0–29.0]	2.406	0.016*
MoCA	23.0 [20.0–25.3]	25.0 [23.0–27.0]	4.573	0.000*
Stroop1	29.5 [26.0–36.0]	27.0 [24.0–32.0]	3.075	0.002*
Stroop2	49.0 [38.0–57.0]	43.0 [35.8–50.0]	3.446	0.001*
Stroop3	84.5 [73.0–107.5]	79.5 [69.0–97.0]	2.222	0.026*
Boston naming	25.0 [23.0–27.0]	26.0 [24.0–28.0]	2.601	0.009*
AVLT-N1	3.0 [2.0–4.0]	3.0 [2.9–4.0]	1.947	0.051
AVLT-N2	5.0 [4.0–6.0]	5.0 [4.0–7.0]	1.869	0.062
AVLT-N3	6.0 [5.0–7.0]	6.0 [5.0–8.0]	2.377	0.017*
AVLT-N4	4.0 [2.1–6.0]	5.0 [3.0–6.0]	3.486	0.000*
AVLT-N5	4.0 [2.8–6.0]	5.0 [3.0–7.0]	2.020	0.043*
AVLT-N6	4.0 [2.0–6.0]	4.0 [3.0–7.0]	1.628	0.104
Clock drawing	4.0 [3.0–4.0]	4.0 [3.8–4.0]	2.178	0.029*
TMT-A	81.4 [61.5–101.4]	68.0 [49.8–87.0]	3.125	0.002*
TMT-B	206.0 [150.8–260.0]	180.5 [141.0–240.0]	2.029	0.042*
Digit span-Forward	7.0 [6.0–8.0]	8.0 [7.0–9.0]	3.489	0.000*
Digit span-Backward	3.0 [3.0–4.0]	4.0 [3.0–4.0]	4.475	0.000*
ROCFT	34.0 [29.9–35.0]	34.0 [32.8–36.0]	3.704	0.000*
Hamilton-D	5.0 [2.0–7.0]	3.0 [2.0–6.0]	3.000	0.003*
Hamilton-A	5.0 [3.0–7.0]	3.5 [2.0–6.0]	2.787	0.005*

CSVD, cerebral small vessel disease; MMSE Mental State Examination; MoCA, Montreal Cognitive Assessment; AVLT, Rey Auditory Verbal Learning; TMT, Trail Making test; ROCFT, Rey Osterrieth complex figure. *$P < 0.05$
